# Seed Endophyte Microbiome of *Crotalaria pumila* Unpeeled: Identification of Plant-Beneficial Methylobacteria

**DOI:** 10.3390/ijms19010291

**Published:** 2018-01-19

**Authors:** Ariadna S. Sánchez-López, Isabel Pintelon, Vincent Stevens, Valeria Imperato, Jean-Pierre Timmermans, Carmen González-Chávez, Rogelio Carrillo-González, Jonathan Van Hamme, Jaco Vangronsveld, Sofie Thijs

**Affiliations:** 1 Centre for Environmental Sciences, Hasselt University, Agoralaan building D, 3590 Diepenbeek, Belgium; ariadnas@colpos.mx (A.S.S.-L.); vincent.stevens@uhasselt.be (V.S.); valeria.imperato@uhasselt.be (V.I.); jaco.vangronsveld@uhasselt.be (J.V.); 2 Laboratory of Environmental Chemistry and Environmental Microbiology, Edaphology, Colegio de Postgraduados, Campus Montecillo, Carretera Mexico-Texcoco km 36.5, Montecillo 56230, Mexico; carmeng@colpos.mx (C.G.-C.); crogelio@colpos.mx (R.C.-G.); 3 Laboratory of Cell Biology and Histology, University of Antwerp Campus Drie Eiken, Universiteitsplein 1, 2610 Wilrijk, Antwerp, Belgium; isabel.pintelon@uantwerpen.be (I.P.); jean-pierre.timmermans@uantwerpen.be (J.-P.T.); 4 Department of Biology, Thompson Rivers University, 950 McGill Road, Kamloops, BC V2C0E1, Canada; jvanhamme@tru.ca

**Keywords:** metalliferous soil, trace metals, *Methylobacterium*, seed core microbiome, plant growth-promoting endophyte, xylem

## Abstract

Metal contaminated soils are increasing worldwide. Metal-tolerant plants growing on metalliferous soils are fascinating genetic and microbial resources. Seeds can vertically transmit endophytic microorganisms that can assist next generations to cope with environmental stresses, through yet poorly understood mechanisms. The aims of this study were to identify the core seed endophyte microbiome of the pioneer metallophyte *Crotalaria pumila* throughout three generations, and to better understand the plant colonisation of the seed endophyte *Methylobacterium* sp. Cp3. Strain Cp3 was detected in *C. pumila* seeds across three successive generations and showed the most dominant community member. When inoculated in the soil at the time of flowering, strain Cp3 migrated from soil to seeds. Using confocal microscopy, Cp3-mCherry was demonstrated to colonise the root cortex cells and xylem vessels of the stem under metal stress. Moreover, strain Cp3 showed genetic and *in planta* potential to promote seed germination and seedling development. We revealed, for the first time, that the seed microbiome of a pioneer plant growing in its natural environment, and the colonisation behaviour of an important plant growth promoting systemic seed endophyte. Future characterization of seed microbiota will lead to a better understanding of their functional contribution and the potential use for seed-fortification applications.

## 1. Introduction

Metal contaminated sites are a threat to human health when left untreated and lead to significant economic costs [1]. In Europe, an estimated 137,000 km^2^ or 6.24% of the agricultural soils is contaminated with trace metals [1]. In China, as much as 10.18% of farmland soil is heavily contaminated and about 13.86% of cereal production is affected [2]. Besides anthropogenically contaminated soils, natural metalliferous soils exist, including serpentine soils (enriched in Ni, Cr, Co) and calamine soils (enriched in Cd, Pb, Zn), which are interesting for mining. However, at the same time these activities destroy the soil structure and life. On natural metalliferous soils and mine tailings (waste heaps), metallophyte plants can be found which are tolerant to high concentrations of trace metals. Some of these metallophytes are able to (hyper)accumulate trace metals in their aboveground tissues in high concentrations without showing any symptoms of toxicity [3,4]. Several mechanisms are at the basis of metal detoxification, including the sequestration of trace metals in the vacuole or apoplast and/or the complexation of metals with metal-binding peptides, such as metallothioneines and phytochelatins [5,6]. In contrast to these metallophytes, most agricultural crops are very sensitive to elevated metal concentrations in soils. Therefore, it is of crucial importance to develop methods to reclaim heavily disturbed metal contaminated sites, and improve plant growth and metal tolerance.

Plants are colonized by an enormous diversity of microorganisms with a range of metabolic functions that allow for soil microorganisms to affect metal uptake, transformation, and accumulation [7,8]. In this respect, rhizosphere micro-organisms have been extensively studied for their interactions with metalliferous plants [8,9]. They were shown to contribute to metal accumulation in plants either directly or indirectly by stimulating plant growth, increasing the surface area of roots, the release of nutrients, and affecting metal uptake, or by (im)mobilizing and/or complexing metals [5,8,9]. Endophytic bacteria are also recognized as very important in respect of stress tolerance and plant growth [10]. Endophytes can promote plant growth and metal uptake directly by producing plant growth beneficial substances, phytohormones, siderophores and specific enzymes, metal mobilizing compounds, and biosurfactants; and, indirectly through controlling plant pathogens or by improving plant stress tolerance by producing 1-aminocyclopropane-1-carboxylate (ACC)-deaminase [10,11]. In contrast to the available amount of information on rhizospheric and shoot endophytic bacteria of metalliferous plants, very little is known about seed endophytes. Mastretta et al. [12] demonstrated that seeds of tobacco grown on a Cd containing growth medium carried beneficial endophytes, which improved biomass production under conditions of Cd exposure, and resulted in higher plant Cd concentrations when compared to non-inoculated plants [12]. Truyens et al. [13] have shown that the seed endophytic community of *Arabidopsis thaliana* exposed to Cd for several generations, contained different bacterial taxa and different functional properties, e.g., metal tolerance and ACC-deaminase dominated in the strains isolated from seeds grown on a Cd enriched growth substrate, while siderophore production, IAA production, and organic acids was more prevalent in endophytes from seeds of plants grown in absence of Cd [13,14]. This suggests that certain endophytes and traits can be transferred to next generations and might be of high importance for seed germination and seedling development.

*Crotalaria pumila* is an annual herbaceous (sub)tropical plant species with wide environmental tolerance. It is a potential accumulator and phytoextractor of Zn, growing on metalliferous soils in the semi-arid region in Zimapan, Mexico. The plant has unique adaptations to deal with metal stress and accumulates up to 300 mg Zn per kg of dry weight (DW) in the shoots, which reflects the total Zn concentrations in the soil [15]. To better understand the abilities of this metallophyte to grow and proliferate under these harsh environmental conditions, we sampled and characterized the seed microbiota over three successive years which led to the interesting discovery of a high abundance of Methylobacteria present in the samples [16]. But so far, we have yet an incomplete understanding of how these Methylobacteria can contribute to plant growth and health. Therefore, we performed a detailed investigation of the seed microbiome of *C. pumila*, in order to figure out which are the dominant re-occurring seed endophytes (seed core microbiome), which representative cultured *Methylobacterium* spp. can be characterized and which plant growth promoting properties they have. In addition, what is the origin of *Methylobacterium* in the seed, is it a systemic endophyte or a stochastic phenomenon. In addition, the effects of inoculation with *Methylobacterium* sp. strain Cp3 on seed germination were evaluated.

This paper describes for the first time the seed core microbiome of *Crotalaria pumila*, and presents the full characterized potential of its dominant colonizer, *Methylobacterium* sp. Cp3. We found that strain Cp3 is able to migrate from sand to seeds, produces a plethora of plant growth promoting (PGP) compounds and has multiple metal resistant elements in its genome. Moreover, it has the potential to perform aerobic anoxygenic photosynthesis and can consume 1C-compounds from plants. Most importantly, strain Cp3 can improve seed germination and significantly increases root radicle length. Future studies on other plant species growing on the same site, such as *Brickellia veronicifolia*, *Dalea bicolor*, *Dichondra argentea*, and *Pteridium* sp., and seed microbiome interaction studies can further develop our knowledge on the importance of seed microbiota of metalliferous plants, and may lead to the development of seeds characterized by improved germination and seedling/plant development on trace metal contaminated sites.

## 2. Results

### 2.1. Seed Microbiome of C. pumila

**Figure 1:**
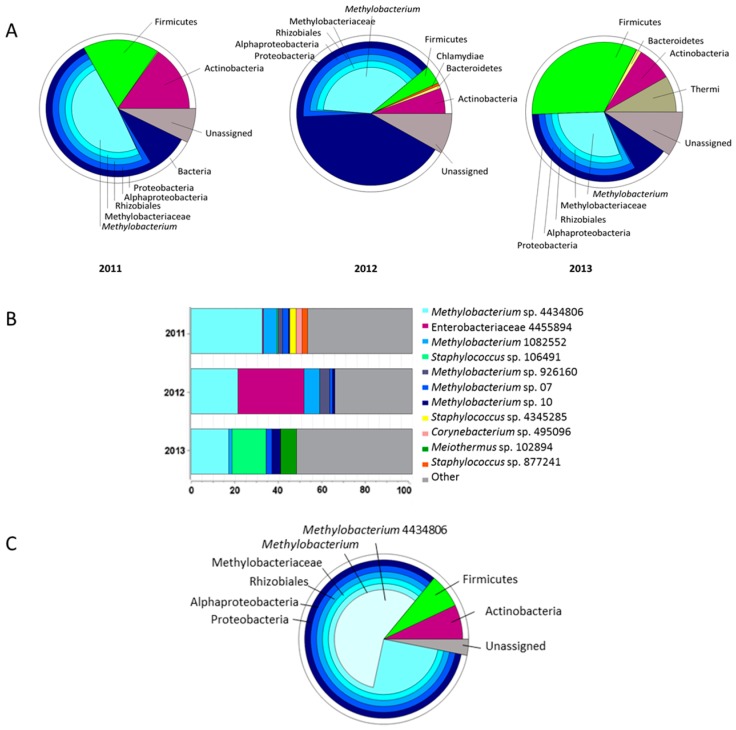
Composition of the core microbiome of *Crotalaria pumila* seeds throughout three consecutive generations. Average of core microbiome in each generation (**A**); and, identification of the most abundant taxon in each generation (**B**) and in the core microbiome over the three generations (**C**).

The seed microbiome of *C. pumila* was identified in seed pods that are collected over three consecutive years. *Methylobacterium* is the most abundant genus of the seed microbiome, constituting 48.90% in 2011, 37.62% in 2012, and 29.91% in 2013 (Figure 1A). In 2012, also Enterobacteriaceae accounted for a large fraction (Figure 1B). In addition to Methylobacteria and Enterobacteriaceae, other dominant taxa identified in the seeds were Firmicutes (Staphylococcus), and Actinobacteria with Corynebacterium. Interestingly, within the Methylobacteria group, a single OTU_4434806 was the most dominant member across all of the years (Figure 1B). A sample per sample comparison showed that this specific OTU occurred in 11 of 12 different seed pods collected. Analyses of the seed core microbiome, defined as the bacterial taxa occurring in at least 50% of the samples over three consecutive years, confirmed that *Methylobacterium* OTU_4434806 was the most abundant OTU (57%) of the seed bacterial community (Figure 1C). Because of the high prevalence of this OTU in the seeds and the potential importance for seed germination and plant growth, we queried our culturable collection for representatives matching at least 99% of the partial 16S rDNA sequence with OTU_4434806. This led to the identification of isolate *Methylobacterium* sp. Cp3, the candidate for studying the plant colonisation mechanism of Methylobacteria.

### 2.2. Endophytic Colonisation of Methylobacterium sp. Cp3 from Soil Substrate to Seed

**Figure 2:**
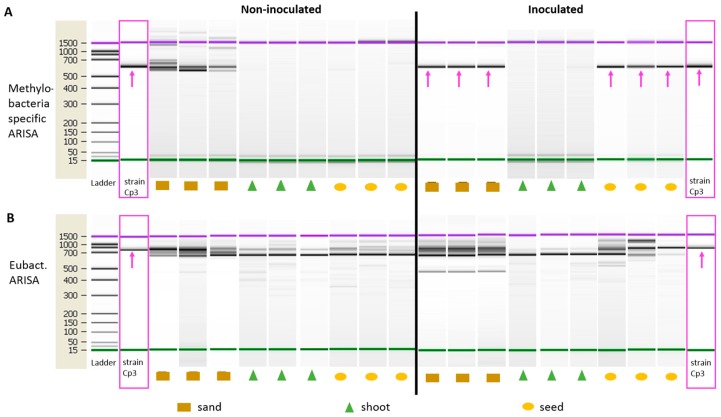
Methylobacteria specific ARISA fingerprints and general bacteria Automated Ribosomal Intergenic Spacer Analysis (ARISA) fingerprints of the control non-inoculated *A. thaliana* plants and plants inoculated with *Methylobacterium* sp. Cp3. Pink boxes indicate the fingerprint of the inoculated strain Cp3 and the arrows point to the specific amplicon for Cp3.

To gain insights in the mode of plant colonisation, strain Cp3 was inoculated via the nutrient solution added to the soil on which *Arabidopsis thaliana* plants were growing. We chose *A. thaliana* for these experiments because of the long generation time of *Crotalaria* (six months), when compared to *A. thaliana* (10 weeks), and moreover, *Methylobacterium* has been shown to be part of the endophytic community *A. thaliana* seeds as well [13]. Control plants were watered with only the nutrient solution. Ten weeks after inoculation, strain Cp3 was found in the seeds by *Methylobacterium* specific Automated Ribosomal Intergenic Spacer Analysis (ARISA)-fingerprint, while the strain was not present in non-inoculated plants (Figure 2A, fragment of 620 bp). Strain Cp3 was also highly abundant in the inoculated soil, while native Methylobacteria species were present in the non-inoculated non-sterile soil, as shown by DNA fingerprints of different sizes (Figure 2A). *Methylobacterium* Cp3 was not detected in the shoot of mature inoculated plants, indicating that at the time of seed ripening and drying of the shoot, the seed is a more conducive and protective habitat. We performed also ARISA using general bacteria primers (Figure 2B). Although this gives a more complicated and rich fingerprint profile, the 750-bp PCR-fragment corresponding to *Methylobacterium* Cp3 could be distinguished in the seeds of inoculated plants, in the soil and also in the shoot, providing an additional confirmation of the systemic spread and the presence of the inoculated strain throughout the plant. Because the general bacteria primers target a broad range of bacteria, amplifying many more fragments not necessarily corresponding to Methylobacteria, we consider the fingerprint profiles with Methylobacteria-specific primers as more confirmative for Cp3 colonisation, while the general bacteria primers provide an indication of the total bacterial community diversity in the seeds, shoot, and sand substrate.

In addition to ARISA fingerprinting, the presence of strain Cp3 in seeds was assessed by counting the abundance of Methylobacteria colony forming units (CFU) on methanol impregnated medium and BOX fingerprint analyses. Inoculated plants were significantly more colonised with 8.6 × 10^5^ ± 1.5 pink-colored CFU g^−1^ seed, against 2.1 × 10^4^ ± 3.5 CFU g^−1^ for seeds of non-inoculated plants. BOX fingerprint profiles of randomly picked colonies confirmed that the inoculated *Methylobacterium* sp. Cp3 indeed was present in surface-sterilised macerated seeds, and that it was alive and actively growing (Figure S1).

### 2.3. Establishment of Methylobacterium sp. Cp3 in Plant Tissue in Presence of Trace Metals

**Figure 3:**
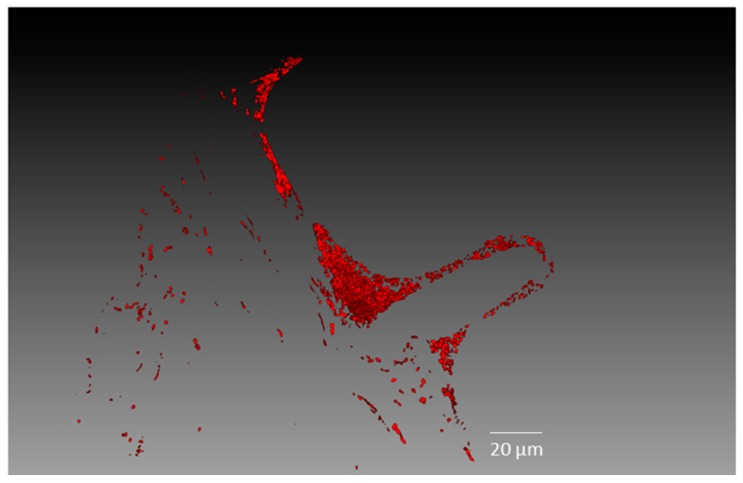
Confocal microscopy picture in the red-channel with mCherry-tagged *Methylobacterium* sp. Cp3 lining the root hair surface of *Crotalaria pumila* in medium supplemented with Zn and Cd.

**Figure 4:**
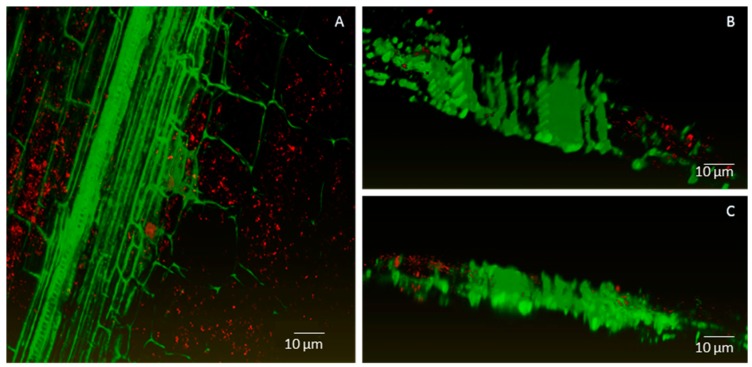
Confocal images of combined m-Cherry fluorescence (red) and plant autofluorescence (green) showing root colonisation by *Methylobacterium* strain Cp3. Maximum intensity projection (**A**) and volume rendering (**B**,**C**) where Cp3-mCherry is localized intracellularly in root cortex of *Crotalaria pumila*. Confocal stack thickness is 58 μm and was acquired with the Ultra VIEW VoX (PerkinElmer, Zaventem, Belgium) using the CFI Plan Apochromat VC objective 20.0 × 0.75. Z-step was 1 μm. Three-dimensional models were created with the software Amira 6.0.1 (FEI software, Hillsboro, OR, USA).

**Figure 5:**
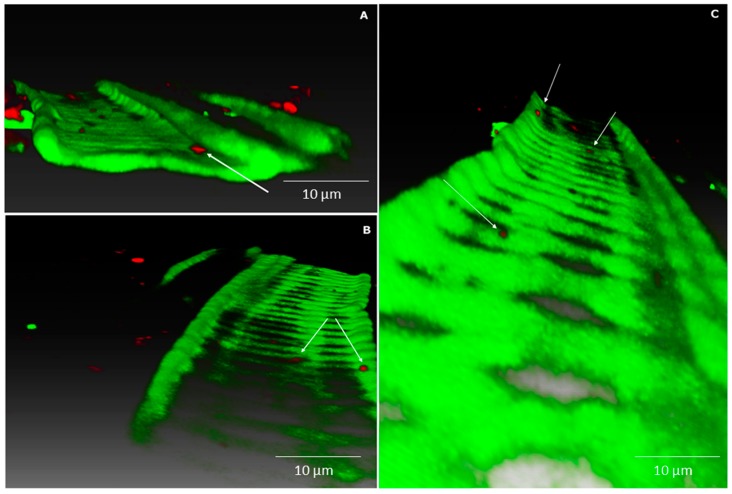
Confocal images with combined mCherry fluorescence (red) and plant autofluorescence (green). Volume rendering (**A**–**C**) of *Methylobacterium* sp. Cp3-mCherry colonising the xylem vessels in the stem of *Crotalaria pumila* growing in medium supplemented with Zn and Cd. White arrows indicate strain Cp3. Confocal stack has a thickness of 54 μm, acquired with a Ultra VIEW VoX (PerkinElmer) using the CFI Plan Fluor objective 40.0 × 0.75. Z-step was 1 μm. Three-dimensional models were created with the software Amira 6.0.1 (FEI software, Hillsboro, OR, USA).

Confocal laser scanning microscopy was used to study where and how strain Cp3-mCherry is colonising *C. pumila*. As can be observed from Figure 3, radicles of inoculated seeds were intensively colonised by Cp3 10 days after inoculation when exposed to the trace metals Cd and Zn. More specifically, a biofilm of bacterial cells can be observed on the root surface and root hairs (Figure 3). Interestingly, tagged Cp3 also was detected as endobacterium inside root cortex cells in extreme dense colonisation (Figure 4A–C). In addition, xylem vessels were colonised with lower numbers of bacteria, mainly solitary cells, as demonstrated using three-dimensional (3D)-volume rendering (Figure 5). During these microscopic analyses, higher numbers of tagged cells were observed in roots in comparison to stems. This was confirmed after isolating tagged cells from surface sterilized plant tissues (Figure S2). The number of CFU of mCherry-*Methylobacterium* sp. Cp3 cells in roots was 3.2 × 10^6^ ± 0.1, in comparison to 0.17 × 10^6^ ± 0.01 in stems of *C. pumila*.

### 2.4. Strain Cp3 Inoculation Improves Seed Germination and Plantlet Survival under Cadmium Stress

**Figure 6:**
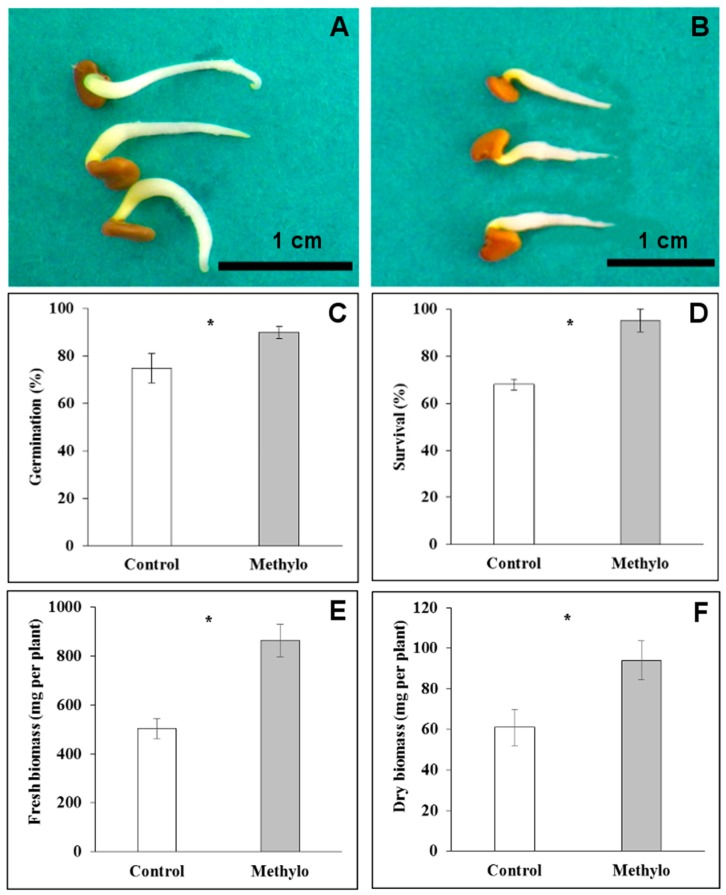
Control seedlings of *Crotalaria pumila* (**A**) and seedlings inoculated with *Methylobacterium* sp. Cp3 (**B**) at five days after start of germination. Percentage of germination (**C**); survival rate (**D**); and, fresh (**E**) and dry biomass (**F**) of inoculated and non-inoculated plants. * significant difference between treatments according to *t*-test (*p* < 0.05), mean ± standard deviation.

To assess whether strain Cp3 influences the germination of *C*. *pumila* seeds, surface-sterilised seeds were inoculated with 10^6^ CFU mL^−1^. This treatment resulted in a significantly higher germination rate in the inoculated seeds (90 ± 2.6%) when compared to the control (75 ± 6.3%) (*t*-test, *p* < 0.05, *n* = 50). Moreover, at five days after the start of germination, radicles of inoculated seedlings were significantly longer (1.8 ± 0.3 cm) than radicles of non-inoculated seedlings (1.2 ± 0.12 cm) (*t*-test, *p* < 0.05, Figure 6). To assess whether colonisation by Cp3 also protects the plantlets against Cd and Zn stress, inoculated and non-inoculated seeds were sown on a trace metal contaminated soil. After 10 days, the percentages of survival were determined: strain Cp3 inoculated seedlings had a significantly higher survival rate (95 ± 4.8%) than those that were not inoculated (68 ± 2.3%). At the end of the experimental period (60 days after transplantation), the inoculated plants produced higher amounts of both fresh and dry biomass (863 ± 66.3 and 94 ± 9.7 mg per plant, respectively) than plants without inoculation (503 ± 42.6 mg of fresh and 61 ± 8.9 mg of dry biomass), demonstrating that strain Cp3 indeed has protective effects for plants under trace metal exposure (*t*-test, *p* < 0.05).

### 2.5. Genes Related to Cadmium Tolerance, Plant Colonisation and Plant-Growth Promotion

**Figure 7:**
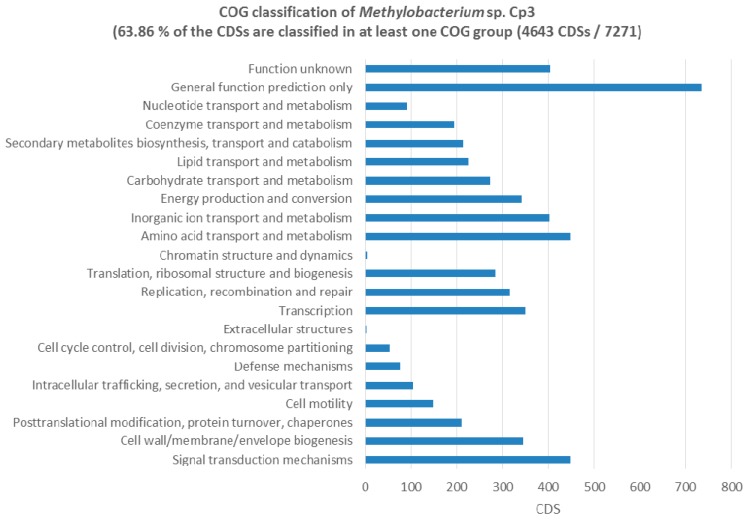
COG classification of the CDS of *Methylobacterium* sp. Cp3.

In the 5.72 Mb draft genome of *Methylobacterium* sp. Cp3, several genes that were related to plant-growth promotion were found including IAA production, acetoin production, and ACC-deaminase (ACCD). The presence of two homologues of indole acetamide hydrolase suggest that the biosynthesis of auxin occurs via the indole-3-acetamide pathway. A homologue of the butanediol-dehydrogenase enzyme (*adh*) was present, and in vitro tests confirmed the production of (R,R)-2,3-butanediol via (R)-acetoin, both important volatile plant hormones. One gene coding for ACCD was detected, which could improve plant growth under stress conditions. In vitro assays for these PGP traits proved that the genes are functional and can be expressed. Furthermore, the strain Cp3 genome contains three copies of the leucyl aminopeptidase (*pepA*) involved in seed germination protein turnover. Several genes with a role in carbohydrate metabolism were found (272 CDS, Figure 7), including genes involved in xylan degradation, cellulose degradation, d-mannose, galactose, glucose, glycogen, l-arabinose, trehalose, and xylose degradation; these sugars are important components in plant seeds. Also Biolog GN2 plates inoculated with strain Cp3 confirmed the utilisation of d-mannose, d-galactose, d-glucose, glycogen, l-arabinose, and d-trehalose in addition to the use of methyl pyruvate, and carboxylic acids like acetic acid, formic acid, β- and γ-hydroxybutyric acid, α-ketogluaric acid, d,l-lactic acid, malonic acid, propionic acid and succinic acid, the amide succinamic acid, and the amino acids, and l-asparagine and l-aspartic acid. Amino acid transport and metabolism account for a total of 447 CDS of the annotated ones in the genome (Figure 7). Interestingly, also several genes coding for enzymes that are involved in superoxide radical degradation are present including five catalases and two superoxide dismutases *sodB* and *sodC*. Strain Cp3 also possesses 24 genes involved in metal tolerance, such as copper resistance protein CopZ, and several metal transporters, including metal ABC permeases, heavy metal RND transporters in addition to resistance and binding proteins, arsenate reductase, and arsenite oxidase operon. Plating on minimal medium supplemented with high concentrations of trace metals showed that Cp3 is tolerant to 2 mM Cd, 5 mM Zn, 0.5 mM Pb, and 0.4 mM Cu. Furthermore, genes coding for the methanol dehydrogenase and methanol oxidation system are present, which allows the strain to use methanol, an 1-C compound, as sole source of energy. Genes that encode for proteins involved in photosynthesis were located, including bacterial light-harvesting complex (one gene), photosynthetic reaction center (two genes), as well as biosynthesis of chorophyll (four genes), bacteriochlorophyll (10 genes), and carotenoids (10 genes). To confirm the presence of the photosystems we recorded the absorbance and emission fluorescence spectra of strain Cp3 grown under light and dark regime. We observed a strong absorbance at 360 nm, and using this wavelength as excitation, we recorded several emission peaks, one at 450, at 520, and 650, and a smaller one in the near infrared 820 nm (Figure S3). These wavelengths can correspond to bacteriochlorophyll and the ability to perform aerobic anoxygenic photosynthesis.

**Figure 8:**
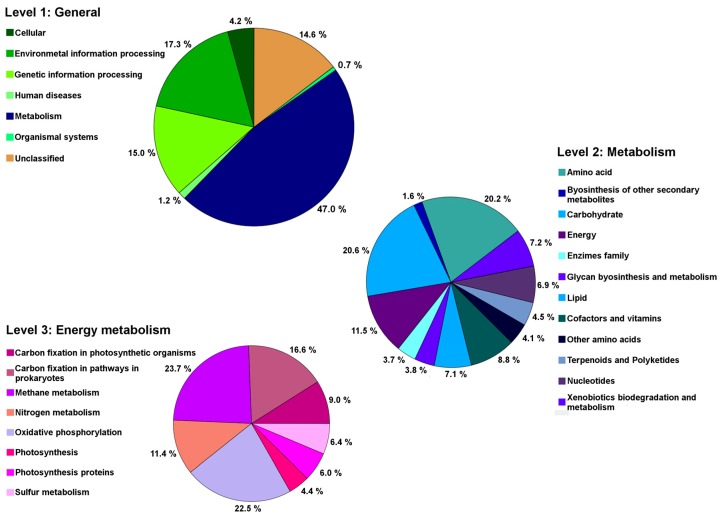
Predicted metabolic functions of *Methylobacterium*, the dominant member of the seed endophyte core microbiome of *Crotalaria pumila* across three generations.

*Methylobacterium* Cp3 is a dominant member of the *C. pumila* seed endophyte community, so its genes and genomic content potentially contribute significantly to the total seed microbiome function. Hence, we used Phylogenetic Investigation of Communities by Reconstruction of Unobserved States (PICRUSt) to explore the metagenome functional content of *C. pumila* seeds. Metabolic functions of bacterial 16S rRNA genes in *Methylobacterium* were the most abundant and predicted for 47.0% of the genome (Figure 8). Within these functions, at level 2, the most dominant processes were related to carbohydrate metabolism (20.6%), amino acids (20.2%), and energy metabolism (11.5%), with the latter possessing genes coding for enzymes involved in carbon fixation, methanol and nitrogen metabolism.

## 3. Discussion

In this study, we examined the seed endophyte microbiome of *C. pumila* and investigated the manner of plant colonization of a representative *Methylobacterium* sp. (strain Cp3), in addition to the study of its ability to influence seed germination and seedling growth. The systemic approach followed here, from characterization of the seed microbiome in the field using 454 pyrosequencing, to in plantae colonization tests under non-sterile conditions, and in gnotobiotic conditions using confocal microscopy, seed-inoculation experiments, and genetic and phenotopytic characterization of strain Cp3, allowed for an integrated and holistic picture of the seed endophyte microbiome of *C. pumila* and the origin, colonisation, and behavior of one of its predominant seed endophytes.

The results showed the steps of the colonisation by strain Cp3: entrance from the sand substrate into plant root tissues, migration to the aboveground plant tissues through the xylem vessels, and finally establishment in the seeds. This process of plant colonisation by endophytes has been suggested previously by Compant et al. [17] and Truyens et al. [18]. However, in this work, the mentioned process was demonstrated taking into account conditions of metal exposure. Although other seed endophytes of *Crotalaria* could be studied more in detail, *Methylobacterium* attracted our attention because it was a highly abundant taxon in the seeds of *C. pumila* growing on a mining site, and was consistently present over three seed generations. Hence, we hypothesize that this microorganism plays an important role for the plant, for example in protecting young seedlings from metal stress. In other studies, *Methylobacterium* has been reported as a coloniser of plant leaf surfaces, but also as an endophyte of diverse plant species growing on metal containing substrates, which might confirm our hypothesis [19,20,21].

The core microbiome of a plant is considered as being a group of microorganisms shared among plants of a population under study; changes occurring over time should be taken into account to define the core microbiome [22]. In this work, we defined the seed core microbiome of *C. pumila,* taking into account both aspects, a population of plants colonising metal ore mining residues and changes across three consecutive years (Figure 1). *Methylobacterium* was found as the taxon dominating the seed core microbiome of this pioneer plant species colonising metal-contaminated mine residues (Figure 1). The structure of the core microbiome suggests that they are not random guests in the plant habitat; they seem to play essential roles, interacting with the plant host, and influencing plant physiology, as suggested by Gaiero et al. [23]. Especially the seed endophytic microbiome can be considered to be a key player during the acclimatisation to local conditions [22]. Recently, using 16S rRNA gene amplicon sequencing of seed samples, Truyens et al. [14] identified a small subset of the *A. thaliana* seed microbiome that is conserved across generations. Functional traits of the microbiome (IAA production and ACCD activity) were found to be more important than genotypes for subsequent bacterial seed community composition [14].

According to the results obtained in this work, the core microbiome of *C. pumila* seeds holds genes that are related to nitrogen fixation, photosynthesis, and methanol metabolism (Figure 8). We observed genes for nitrogen fixation in our strain, previously others have described that Methylobacteria contain nitrogen-fixation related genes which might contribute to ammonium provision to its host plant [24]; therefore, strain Cp3 can supply nitrogen to its host plant *C. pumila* when growing on metal-contaminated mine residues. The presence of methanol metabolism related genes, especially in the case of *Methylobacterium* (Figure 8), represents a potential advantage for the core microbiome. During plant colonisation, methylotrophic endophytes can take advantage of the methanol that is released by plants and use it as an additional energy source [19]. Therefore, colonisation of the host plant is more efficient in comparison to other plant-associated bacteria [25]. In turn, the core microbiome provides beneficial plant interactions; Abanda-Nkpwatt et al. [26] demonstrated that *M. extorquens* strains can sustain themselves using the methanol released by the host plant and simultaneously improve the growth of the seedlings. The prediction of functions indicated that Cp3 can perform aerobic anoxygenic photosynthesis, and this is in line with earlier reports that mentioned that *Methylobacterium* contains core genes that are related to photosynthesis, including those encoding for the light-harvesting complex [27], and those involved in the synthesis of bacteriochlorophyll and carotenoids [28]. Some authors mentioned that the carotenoid pink pigment, which is characteristic of Methylobacteria, is also associated with resistance to reactive oxygen species and UV light [29,30,31]. As a dominant member of the core microbiome (Figure 1) and taking into account the functional potential of *Methylobacterium* (Figure 8), it is expected that this methylotrophic seed endophyte provides its host plant multiple plant growth promotion benefits during germination and growth on metal mine residues. These findings, methylotrophic metabolism, involvement in phytohormone synthesis, metal-tolerance, and photosynthesis, may explain the relatively high abundance and transmission of *Methylobacterium* in *C. pumila* seeds and the success of both, the endophyte and its host, in harsh conditions.

Results obtained in this work demonstrated that *Methylobacterium* sp. Cp3 can be transmitted from a soil substrate to the seeds (Figure 2). The soil, besides providing nutrients, water, and imposing potential stressors to plant growth (in this case metals), is also an important reservoir for endophytic bacteria, including seed endophytes, which are recruited from this pool [32,33,34,35]. Previously, it has been reported that Methylobacteria can colonise roots of different plant species [36,37], and also stems and leaves [38,39]. As also mentioned by Araújo et al. [40], the initial step in the colonisation process seems to be the formation of biofilms on roots and on root hairs. Subsequently, the entrance into root cells occurs, as it has been demonstrated, that proliferating root hairs and side roots are important entry points for endophytic colonisation [41,42,43]. In this study, we present evidence that *Methylobacterium* sp. Cp3 not only colonises roots (Figure 3), and thereby protects *C. pumila* locally from stress when growing in a metal-contaminated substrate, but that the studied strain spreads systemically through the plant through the xylem (Figure 4 and Figure 5) and also ends up in the seeds in order to protect future generations of *C. pumila* plants from metal toxicity.

As a mutualistic symbiont, *Methylobacterium* sp. Cp3 used in this work, was shown to be tolerant to metals (5 mM Zn, 2 mM Cd, 0.4 mM Cu), able to solubilize phosphate and to produce plant hormones (IAA) (which can promote plant growth), and to produce ACCD (which can decrease stress of their host) [44]. However, it remains to be investigated whether *Methylobacterium* sp. Cp3 possesses anti-fungal properties or can act as a bio-control agent in *C. pumila* seeds, besides protection against metal-stress and promotion of plant growth. Altogether, the functional analyses suggest that Methylobacteria possess traits that assist them to survive in the metal contaminated mine residues in the semi-arid area of Zimapan (Mexico), and have multiple traits to help plant growth and development. Therefore, from a plant perspective, the most tolerant bacterial phenotypes are primarily recruited from the original environment from which microbes can be selected.

It has to be noted that despite the participation of seed endophyte Cp3 in the establishment of its host plant, the mechanisms of metal tolerance of the plant itself still remain unclear. Since *C. pumila* is reported to possess high antioxidant activity [45,46], it is logical to think that such characteristics might result helpful in a metal contaminated environment as well. However, this mechanism is studied only from a medicinal point of view, the antioxidant activity of *C. pumila* in metal tolerance can be investigated in the future.

To the best of our knowledge, the present study is the first in which a seed endophyte was observed colonising the stem xylem vessels in the presence of metal stress as environmental variable (Figure 5 and Figure 6), suggesting that the exposure of the host plant to metals promotes the migration of specific endophytes to the aboveground plant parts. In several other studies, bacterial strains isolated from the rhizosphere [47,48], and some endophytes from stems [49,50,51] and seeds [52,53,54] were shown to colonise the internal root and shoot tissues (including cortex and xylem) under control conditions. Using gfp-tools and under metal exposure conditions, Zhang et al. [55] reported that labelled root endophytes of *Sedum alfredii* (*Burkholderia* and *Variovorax*) were observed inside the root cortex, but no colonisation of plant vascular system was reported. In addition, according to the criteria of a real endophyte, as defined by Schulz and Boyle [56], we conclude that *Methylobacterium* sp. Cp3 is a real endophyte. It was originally isolated from surface sterilised seeds of *C. pumila*, inoculated to plantlets of its host plant species, it was observed colonizing inner plant tissues (Figure 4, Figure 5 and Figure 6) and could be re-isolated (Figure S2).

Both plant species used during this study, *C. pumila* [16,44] and *A. thaliana* [13,14], have been shown to contain endogenous endophytic Methylobacteria, which were able to survive strong seed sterilisation. We found that three times inoculation of *Methylobacterium* sp. Cp3 at the roots during the flowering phase, is effective to enrich seeds with desired *Methylobacterium* strains, and importantly without causing any harm to the host plants. Our results imply that the soil is an adequate route to augment bacteria in seeds, which might have important consequences for geographic influences on seed stock production, crop growth, and exploiting this property for generating bacteria-fortified seeds. Because soils are much richer in bacteria, the importance of the stage of inoculation will have to be investigated more in depth in forthcoming studies. Migration kinetics during seed development should be investigated too. Moreover, the transfer of endophytes from seeds to soil was recently demonstrated in the case of maize [57]; thus, inoculation of a substrate by endophytes from germinating seeds can occur, and endophytes may be recruited by neighbouring plants. This process is highly important when studying colonization of neglected or contaminated soils by pioneer plant species.

To date, endophytes via flower pathway and exogenous seed coating [58,59], are in use to produce better seeds [60,61] and to reduce the incidence of pathogens and pests [62,63]. In our study, the seeds of *C. pumila* naturally enriched with *Methylobacterium* can be an important resource for endophyte-enhanced phytoremediation and the reclamation of metal contaminated soils, as well as inoculant for agricultural crops growing on metal contaminated soils. The developments in new generation sequencing technologies and declining prices have already enabled the study of transgenerational seed-endophyte association in maize [52], wheat [64], and *A. thaliana* [14]. A better knowledge about the overall natural plant associates (diversity, function, and interactions) is thus a good starting point to explore features of other seed endophytes, and intrinsic interactions of plants and their associated microbiomes.

## 4. Materials and Methods

### 4.1. Crotalaria pumila Seed Core Microbiome Analyses

The generation of the 16S rRNA gene amplicon pyrosequencing data used for the core seed microbiome analyses was described in our previous study [16]. The data are available under accession number SRP080874 (GenBank, NCBI, https://www.ncbi.nlm.nih.gov/sra/SRX1998247[accn]). Briefly, closed pods of *C. pumila* were collected from field conditions from at least 40 different individual plants, in laboratory pods were open and unripen and damaged seeds were eliminated. Then, seeds were surface sterilized by washing with phosphorus free detergent and tap water, immersion in NaClO 0.1% solution supplemented with 0.1% Tween 80 for 10 s, and finally rinsed in sterile deionized water (8 × 100 mL). Sterilization was confirmed by plating 100 μL of the last rinsing water on solid medium [16] and by running a PCR on the last rinsing water [14]. Genomic DNA used for the pyrosequencing was extracted from 150 mg of surface sterilized *C. pumila* seeds, and then subjected to PCR reactions using primers 799F and 1391R, according to the conditions reported previously [16].

The *C. pumila* seed core microbiome was determined using Quantitative Insights Into Microbial Ecology (QIIME) and was defined as the phylotypes consistently present in at least 50% of the samples (*n* = 12 samples, four per year) across three consecutive seed generations. Gene prediction with Phylogenetic Investigation of Communities by Reconstruction of Unobserved States (PICRUSt) was applied to predict the functional content from the 16S rRNA dataset of the core microbiome [65]. Function predictions were categorized on the Kyoto Encyclopedia of Genes and Genomes (KEGG) classification.

### 4.2. Methylobacterium Cp3 Isolate Sanger Sequence

*Methylobacterium* Cp3 was previously isolated from *C. pumila* seeds [44]. Blast search was performed using 16S rRNA gene Sanger sequences of Methylobacteria isolates and the 454 pyrosequencing data, to determine the closest culturable representative of Methylobacteria in the seed microbiome using Blast after sequences were quality trimmed and aligned in Geneious v 4.8.5. The 16S rDNA gene sequence of *Methylobacterium* sp. Cp3 was Sanger sequenced before and the sequence was deposited in Genbank with accession number KX056917.

### 4.3. Plant Colonisation Experiment

A pot experiment was performed using *A. thaliana* (ecotype Columbia-0) and *Methylobacterium* sp. Cp3. Seeds were sown in pots with quartz sand (particle size of 0.4–0.8 mm) in the greenhouse at 22/18 °C day/night temperature, with a photoperiod of 14 h, relative humidity of 60%, and plants were supplied regularly with 1/10 diluted Hoagland nutrient solution [66]. At the moment of appearance of the inflorescence stem, the plants were inoculated for the first time with *Methylobacterium* sp. Cp3. To avoid contact with, and thus inoculation of, the inflorescence 2 mL of an exponentially grown *Methylobacterium* sp. Cp3 culture (10^9^ cells mL^−1^ of Hoagland solution) were added carefully with a pipet to the root zone of each plant. Subsequently, once a week for the next two weeks, the plants received a second and third inoculation in the same way as the first one. Aracons with plastic transparent flower sleeves were placed over single plants at the time of inoculation, so that all plant inflorescences were maintained within a sleeve to avoid cross-pollination. When flowering was complete, no more Hoagland solution was given. Seeds were harvested when the soil and plant inflorescence were dry. The seeds were shaken off the plants in a bag, and sieved to separate them from the chaff. Non-inoculated control plants were grown under the same conditions but instead of bacterial inoculum, they were watered with the same amount of 1/10 Hoagland solution. The experiment was performed with 50 plants per condition.

### 4.4. Seed Sample Preparation and DNA-Extraction

*Arabidopsis thaliana* seeds were rinsed and surface sterilised prior to DNA-extraction to remove surface contamination. Briefly, 300 mg of seeds (per sample) were washed for 1 min in sterile deionized H_2_O followed by 1 min in 1% NaClO, and 5 × 5 min in sterile deionized H_2_O. Subsequently, the surface sterilized seeds were homogenized in 2 mL sterile 10 mM MgSO_4_ using a mortar and pestle, and frozen at −80 °C until DNA-extraction. DNA was extracted using the Invisorb Spin Plant Mini Kit (Invitek, Berlin, Germany). In the first step, two stainless steel beads were added to each sample and ground using the Retsch Mixer Mill MM400 (Retsch, Haan, Germany) for 2 × 1 min at maximum frequency (30/s). Subsequently, lysis buffer was added to the homogenised samples, and the standard protocol according to manufacturer’s instructions was followed thereafter. For each condition, five biological replicates were prepared.

### 4.5. Specific Automated Ribosomal Intergenic Spacer Analyses (ARISA)

To monitor the fate of *Methylobacterium* sp. Cp3 in *A. thaliana* tissues during colonisation, *Methylobacterium*-specific ARISA was used. For the PCR assay, primer 1319fGC20 (5′ GCC CCC CGC CCC CGC CGC CCA CTC GRG TGC ATG AAG GCG G 3′) and 45r (5′ GAC GGG ATC GAA CCG ACG ACC 3′) were used [67]. The primers amplify a variable PCR product from a partial fragment of the 16S rRNA gene and the 16S–23S intergenic spacer region as the 45r reverse primer binds to *tRNAALA* gene, upstream of the 23S. The generated PCR products have different lengths for different species, between 440 and 810 bp [67], which allows for the discrimination of the inoculated *Methylobacterium* sp. Cp3 strain and resolving the overall *Methylobacterium* community composition. In addition to *Methylobacterium*-specific ARISA, the bacterial 16S–23S ITS DNA was also amplified with general bacteria primers S-D-Bact-1522-b-S-20 (5′ TGCGGCTGGATCCCCTCCTT 3′) and l-d-bact-132-a-A-18 (5′ CCGGGTTTCCCCATTCGG 3′) [68]. Briefly, each PCR reaction contained 1× High Fidelity PCR-buffer (Invitrogen, Carlsbad, CA, USA), 2 mM MgSO_4_, 0.2 mM of each dNTP, 0.2 µM of each primer, and 1 µL of DNA (1–10 ng per µL), in 25 µL total reaction volume. Cycling conditions for the general bacteria primer pair consisted of a hot start at 94 °C for 3 min and 35 subsequent cycles consisting of 94 °C for 1 min, annealing at 55 °C for 30 s, elongation at 72 °C for 1 min, and final elongation step at 72 °C for 5 min. Amplification conditions for the *Methylobacterium* specific primers were similar, except that an annealing temperature of 69.5 °C was used, after six cycles of touchdown from 72 °C with a 0.5 °C temperature decrease for each cycle.

Amplified reaction mixtures were loaded onto DNA-1000-chips that were prepared, according to the manufacturer’s recommendations, and size sorting of the PCR amplicons was performed on an Agilent 2100 Bioanalyzer (Agilent Technologies, Santa Clara, CA, USA). Expert Software (Agilent Technologies) was used to digitalize the ARISA fingerprints, resulting in electropherograms in ASCII formats that were processed using the StatFingerprints package [69], in the 2.13.0 version of the R project (The R Foundation for Statistical Computing, Vienna, Austria). Peaks of the same size were grouped together. Correct assignment of the peaks was verified by visual inspection of the chromatograms. The manually corrected procedure allowed deciphering whether peaks of different samples, correctly matched the *Methylobacterium* sp. Cp3 unique fragment.

### 4.6. Isolation of Methylobacteria and BOX-Fingerprint Analysis

The seeds from the inoculated and non-inoculated control *A. thaliana* plants were surface sterilized during 0.5 min in 0.1% NaClO supplemented with 0.1% Tween 80 and rinsed thoroughly in sterile deionized water. Seed endophytes were isolated by crushing 50 mg seeds in 500 μL 10 mM MgSO_4_. Dilutions of 0 to 10^−2^ were plated onto 284 medium [70], and incubated in the presence of 1% methanol in a gas-tight containers during 1 week at 25 °C. For each pink-pigmented colony appearing, six representative colonies were chosen for BOX-fingerprint analysis. Briefly, DNA was extracted using the DNeasy 96 Blood and Tissue Kit (Qiagen, Venlo, The Netherlands). A BOX-PCR was used to generate length-variable PCR products using the protocol of Weyens et al. [70]. Fingerprints were visualised under UV illumination on a gel with 1.5% agarose, and gel red nucleic acid stain. Fingerprint band patterns of the isolated colonies and *Methylobacterium* sp. Cp3 were compared through visual inspection.

### 4.7. Confocal Microscopy Analyses

Strain Cp3 was equipped with the mCherry plasmid [71] using a triparental conjugation with *E. coli* DH5A as donor strain, and *E. coli* PRK 2013 as helper bacterium. Briefly, the three strains were mixed in equal volumes and were pipetted on a 0.4 µm Isopore™ membrane filters (Millipore, Billerica, MA, USA) on an LB medium plate. After overnight incubation, the cells were washed from the filter and plated on 284 medium with tetracycline. Pink colonies growing on the selective plate were picked up, and checked for fluorescence using a Nikon epifluorescence microscope (Nikon Eclipse 80i, Nikon Instruments Inc., Melville, NY, USA).

*Crotalaria pumila* seeds used for the colonisation assay were collected from metal-contaminated mine residues [15]. In order to increase and homogenize the germination rate, seeds were submerged for 30 min in concentrated H_2_SO_4_ [72], subsequently rinsed eight times with sterile distilled water and were surface sterilized according to Sánchez-López [44]. *Methylobacterium* mCherry-labeled inoculum was grown in 1/10 diluted 869 liquid medium; subsequently, the culture was washed with MgSO_4_ 10 mM, centrifuged at 2000 rpm for 10 min and resuspended in sterile distilled water. 1 mL of inoculum (10^7^ CFU mL^−1^) was spread on the sterile Petri dish on which seeds were germinating. After 72 h, seedlings were sufficiently developed, and were placed to grow in a gnotobiotic system in vertical agar plates (VAP) using 50-fold dilution of Gamborg’s B5 sterile medium and in the presence of 0.2 mM Cd (CdSO_4_) and 0.4 mM Zn (ZnSO_4_) [73]. Two days later, a second inoculation of the roots was performed (1 mL of 10^7^ CFU mL^−1^ solution). Plants were kept on VAP for in total 10 days and exposed.

Sections of the root, root hairs, and stem were hand cut; subsequently, the outer layer was carefully removed to avoid smearing the bacteria from outside. Longitudinal, transversal, and leaned sections were made and placed on glass plates with coverslip. Z stacks (1 μm) of samples were collected using a spinning disk confocal laser microscope Ultra VIEW VoX, PerkinElmer (Zaventem, Belgium). An excitation wavelength of 561 nm (red) was used for mCherry, and 405 (Dapi) for plant cell wall structures. Lenses used for image acquisition included a 40× CFI Plan Fluor lens (numerical aperture of 0.75; working distance of 0.72 mm) and a 20× CFI Plan Apochromat VC lens (numerical aperture of 0.75, and working distance of 0.72 mm). Images were taken using a Hamamatsu C9100-50 camera (Hamamatsu Photonics K.K., Hamamatsu, Japan).

In order to verify the endophytic colonisation, mCherry tagged bacterial cells were isolated from surface sterilized root and stem sections. Three 1 cm long segments of both roots and stems, from seedlings grown on VAP with and without metals were surface sterilized, as follows: 30 s immersion in 70% (*v*/*v*) ethanol solution, then 1 min in 1% active chloride solution supplemented with Tween 80 (1 droplet per 100 mL solution), and rinsed eight times with sterile distilled water. To verify the effectiveness of the sterilization protocol, 100 µL of the last rinsing water was plated on 1/10 diluted 869 medium. Subsequently, tissues were crushed using a sterile mortar and pestle containing 3 mL of 10 mM sterile MgSO_4_. 100 µL aliquots of the suspension and from the 1/10 and 1/100 dilutions were plated on 1/10 diluted 869 medium supplemented with 0.4 mM Cd and 0.5 mL of tetracycline per liter of medium (10 mg tetracycline mL^−1^ of methanol). The plates were incubated at 28 °C for two days, after which the numbers of CFU were determined.

### 4.8. Image Processing and Three-Dimensional (3D) Visualisation

The confocal pictures were analyzed using ImageJ software and Amira 3D visualisation software version 6.1.0 (FEI Visualisation Sciences Group, Hillsboro, OR, USA). The plant autofluorescence recorded at 405 nm was pseudocolored in green and the mCherry channel (561 nm) was put in red. 3D renderings were created using a volume-rendering visualisation technique (Voltex), based on the emission-absorption of light in every voxel in the stack. Different color maps were then assigned to each channel to distinguish individual fluorescent signals.

### 4.9. In Vitro Plant Growth Promotion Traits Detection

The production of ACCD by strain Cp3 was detected, according to a previously described method [74]. The production of IAA was examined when the strain was grown in a minimal salts medium supplemented with 0.5 mg mL^−1^ tryptophan and was detected by the Salkowski’s reagent reaction [75]. The isolate was screened on National Botanical Research Institute’s phosphate growth solid medium to determine phosphate solubilisation ability [76]. The ability of strain Cp3 to produce siderophores was checked according to the chrome azurol-S assay [77]. Nitrogen-fixing capacity was tested in a semi-solid malate-sucrose medium with bromothymol blue as a pH indicator [78]. The same medium supplemented with 0.12 g L^−1^ NH_4_Cl was used as a positive control. To detect if the isolate produce acetoin the protocol proposed by Romick and Fleming [79] was used. Trace metal tolerance was evaluated by measuring the minimal inhibitory concentration of different heavy metal ions (ZnSO_4_, CdSO_4_, CuSO_4_, NiCl_2_, Pb(NO_3_)_2_) in 284 liquid selective medium pH 7 [70], incubated at 28 °C for five days.

### 4.10. Genome Sequencing, Assembly and Annotation

Strain Cp3 was inoculated into 10 mL of LB medium and grown at 30 °C for 3 days. Cells were collected by centrifugation and used for genomic DNA extraction using the Qiagen Blood and tissue kit (Qiagen) prior to digesting and ligating sequencing adaptors/barcodes using an Ion Xpress Plus Fragment Library Kit (Thermo Fisher Scientific, Waltham, MA, USA). Processed DNA was size-selected (480 bp) on a 2% E-Gel SizeSelect agarose gel and purified using Agencourt AMPure XP beads (Beckman Coulter, Brea, CA, USA). The library dilution factor was determined using an Ion Universal Library Quantitation Kit prior to amplification and enrichment with an Ion PGM Hi-Q Template OT2 400 Kit on an Ion OneTouch 2 System. The enriched Ion Sphere Particles were quantified using an Ion Sphere Quality Control Kit. Sequencing was performed on an Ion 316 Chip v2 (Ion PGM System) with an Ion PGM Hi-Q View Sequencing Kit (Thermo Fisher Scientific). Reads were assembled using SPAdes v3.8.2 (uniform coverage mode; k-mers = 21, 33, 55, 77, 99, 127). Genes were predicted using RAST [80], according to the databases GO, COB, and KEGG. Further annotation was performed using MetaCyc [81] and in the MicroScope platform, under locus tag *Methylobacterium extorquens* Cp3 (METCP3). The draft genome sequence was deposited into NCBI with the accession number MNAO00000000.

## 5. Conclusions

In conclusion, we found that Methylobacteria are important members of the *C. pumila* seed core microbiome and that the plant growth promoting *Methylobacterium* sp. Cp3 can be trans-generationally transmitted, indicating that seeds offer a significant potential for the discovery of vertically transmitted endophytic strains that are important for the growth of next generations of their host plants. *Methylobacterium* sp. Cp3 was able to move from the soil to seeds, when inoculated in the substratum during plant flower development. Interestingly, the studied bacterial strain colonised its host plant via xylem vessels in case of metal exposure through the growth medium and improves seedling growth. Future experiments using a similar approach to identify other systemic endophytes will be valuable tools to increase our knowledge about natural seed endophytes, and whether and how they are vertically transmitted. In addition, further research can focus more on the actively transcribed phenotypic properties of the core microbiome (seed metatranscriptome) and produced compounds (seed metabolome), time-point of entry, and characterising both soil and fungal seed-communities trans-generationally to confirm the links.

## Supplementary Materials

Supplementary materials can be found at www.mdpi.com/1422-0067/19/1/291/s1.
