# Supplementary material for: Seed Endophyte Microbiome of Crotalaria pumila Unpeeled: Identification of Plant-Beneficial Methylobacteria
Source: Int J Mol Sci. 2018 Jan 19;19(1):291. doi: 10.3390/ijms19010291 (PMC5796236; doi:10.3390/ijms19010291)
Supplement: Supplementary file 1 [file ijms-19-00291-s001.pdf]

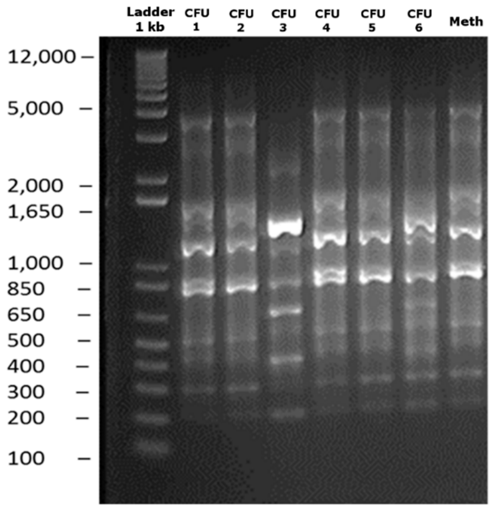

Figure S1. BOX-PCR patterns of Methylobacteria re-isolated from *Arabidopsis thaliana* seeds. Meth = the inoculated *Methylobacterium* sp. Cp3.

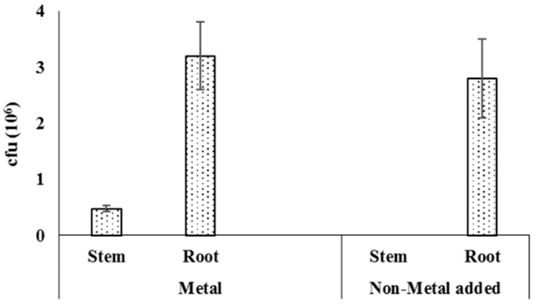

Figure S2. Colonies forming units (CFU) ± SE of mCherry-labeled *Methylobacterium* sp. Cp3 isolated from surface sterilized roots and stems of *Crotalaria pumila*.

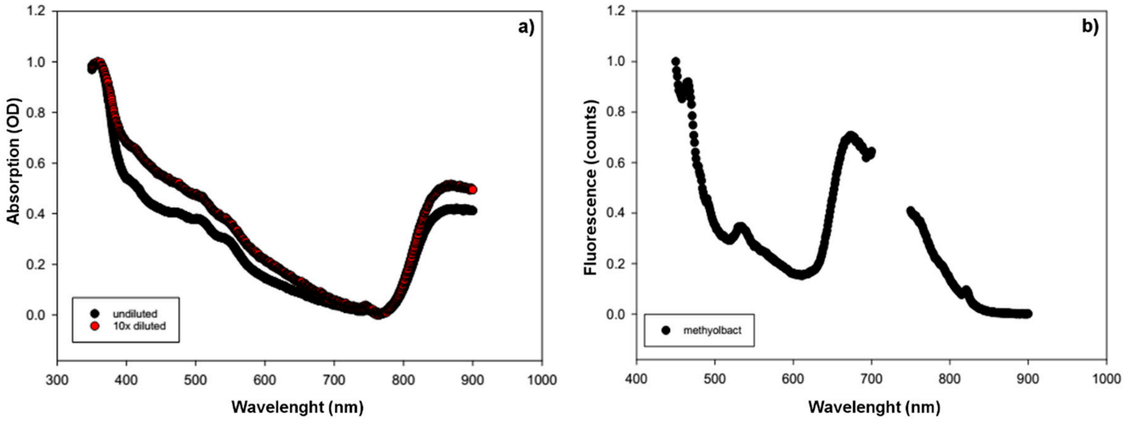

Figure S3. Absorbance and emission fluorescence spectra of strain Cp3 grown under light and dark regime. Strong absorbance was observed at 360 nm (a); emission peaks at 450, 520, 650 and 820 nm (b).
